# POWER OF ATTORNEY FACTORS AND CONDITIONS INFLUENCING ELDER FAMILY FINANCIAL EXPLOITATION

**DOI:** 10.1093/geroni/igad104.0868

**Published:** 2023-12-21

**Authors:** Jennifer Crittenden, Julie Bobitt, Katelyn Galladay, Virginia Vincenti

**Affiliations:** University of Maine Center on Aging, Bangor, Maine, United States; University of Illinois Chicago, Chicago, Illinois, United States; University of Wyoming, Laramie, Wyoming, United States; University of Wyoming, Laramie, Wyoming, United States

## Abstract

Approximately 16% of older adults worldwide experience some form of elder abuse including elder family financial exploitation (EFFE), the unlawful transfer and theft of funds and property perpetrated by a family member. Power of Attorney (POA) arrangements, when constructed and executed properly, can be critical tools for protecting against such exploitation. This national research study examined family reports of EFFE using the Bronfenbrenner Ecological Framework to classify risk factors within the POA arrangement. Interviews were carried out with 31 family members within 24 families that had experienced EFFE. Using constructivist grounded theory analysis methods, the following themes related to risk factors were noted across EFFE stories about professionals involved in the EFFE situations: 1) Conflict of interest between attorneys and POA agents, 2) A perceived lack of competence of consulting attorneys; and 3) A lack of transparency around POA arrangements. Policy and education implications include the need to create legal requirements for transparency reporting and/or registration for POAs, strengthening legal ethics and EFFE training, and providing public education to families about the POA process and how to locate appropriately trained legal professionals.

